# Correction: A scoping review of the residual barriers to skilled birth attendance in Ghana: A conceptual framework and a fish bone analysis

**DOI:** 10.1371/journal.pgph.0003612

**Published:** 2024-08-06

**Authors:** Juliet Abredu, Catherine K. Dwumfour, Boo Alipitio, Mawusi Alordey, Veronica Millicent Dzomeku, Sophie Witter

The images for Figs 1 and 2 are incorrectly switched. The image that appears as Fig 1 should be Fig 2, and the image that appears as Fig 2 should be Fig 1. The figure captions appear in the correct order.

**Fig 1:**
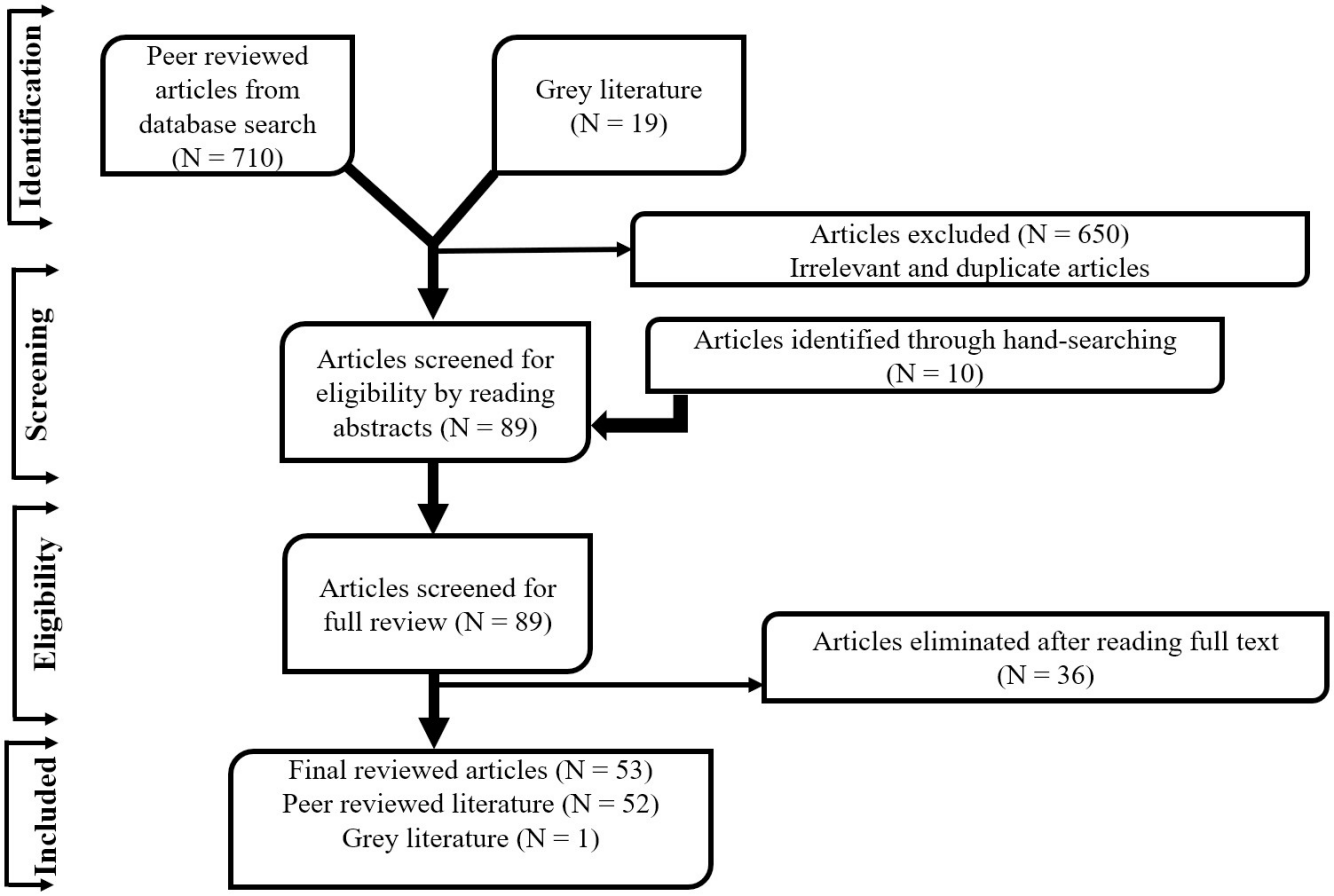
PRISMA-SCR Flow diagram of literature selection. Grey and peer reviewed articles date between 2005 and 2022.

**Fig 2:**
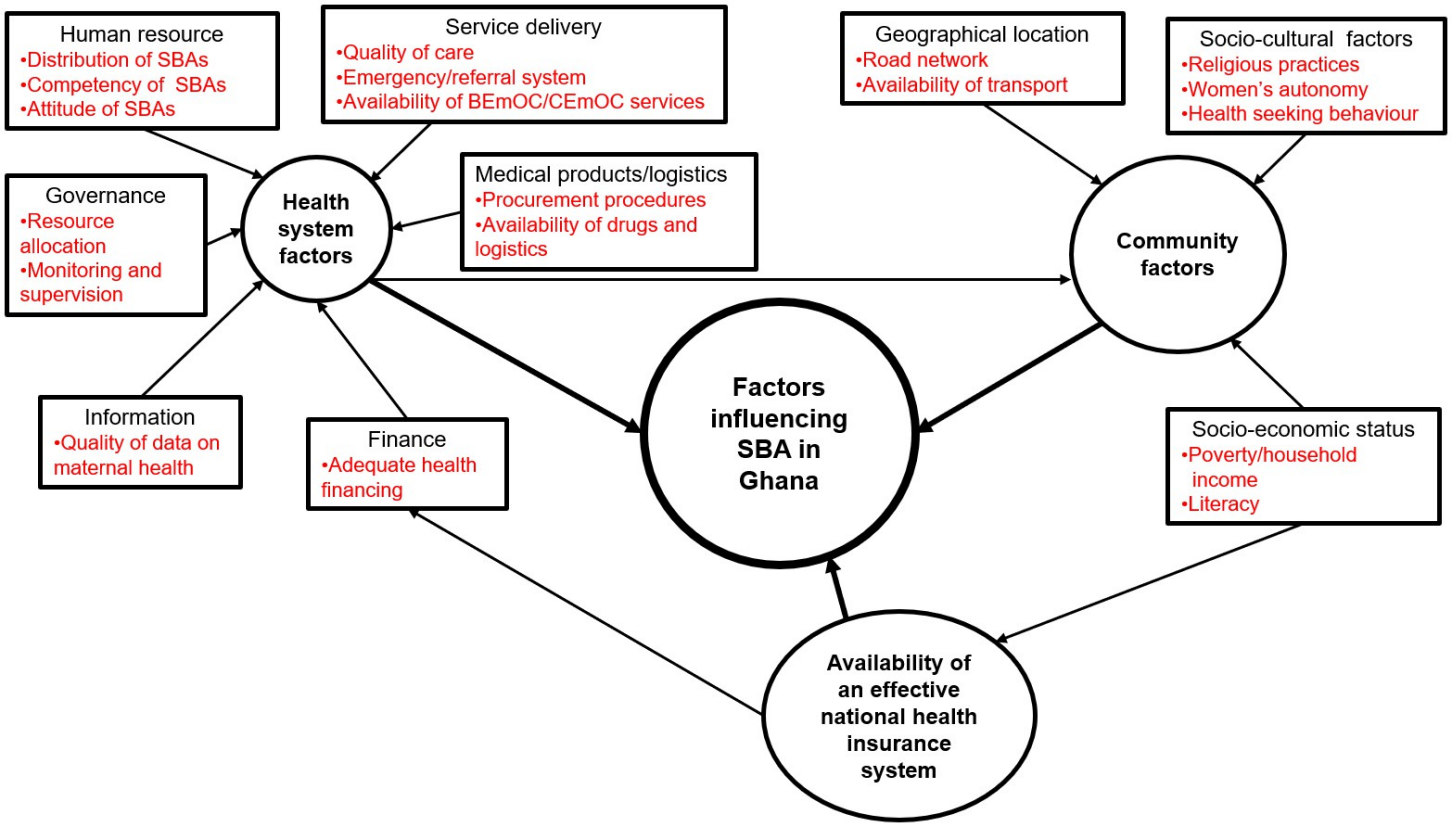
A conceptual Framework of factors influencing SBA in Ghana. The framework shows the interactions of these factors and how they influence SBA in the Ghanaian context.
