# Supplementary material for: A scoping review of the residual barriers to skilled birth attendance in Ghana: A conceptual framework and a fish bone analysis
Source: PLOS Glob Public Health. 2024 Feb 12;4(2):e0002902. doi: 10.1371/journal.pgph.0002902 (PMC10861047; doi:10.1371/journal.pgph.0002902)
Supplement: Attachment — Submitted filename: Response to reviewers.docx [file pgph.0002902.s002.docx]

**Rebuttal Letter**

Dear Dr Chand Chand,

Thank you for the opportunity to submit a revised draft of our manuscript title **“A scoping review of the residual barriers to skilled birth attendance in Ghana: A conceptual framework and a fish bone analysis”** to *PLOS Global Public Health.* We appreciate the time and efforts for reviewing our manuscript and providing valuable feedbacks. We have been able to effect changes to reflect most of the comments and suggestions provided. The changes have been revised in the manuscript shown as track changes.

Please the table below shows a point-by-point response to the reviewer comments and suggestions.

| **COMMENTS FROM REVIEWER 1** | **POINT-BY-POINT AUTHORS’ RESPONSE** |
| --- | --- |
| **Comment 5 a**. *Overall, this was an informative paper. I think it would be helpful for the author(s) to provide a rationale for the wide range of years from which the chosen papers were selected.* | Thank you for your feedback and suggestion.  The papers were chosen from 2005 because, Ghana’s Free Maternal Health Care Policy (FMHCP) was rolled out in all the regions of the country in 2005 and then integrated into the National Health Insurance Scheme in 2008. Also, for the purpose of getting current information on the topic, articles published up to the year 2022 were included. This is included in the manuscript (page 5 lines 102-105). |
| **Comment 5 b:** *It would also be helpful to make sure that the full names of all organizations that are referred to using acronyms are included.* | We acknowledge this comment and revised all sections in the following lines; 89 & 90, 147, 181, 268, 316. |

All grammatical and typographical errors in addition to comments pertaining to meeting the specific submission guideline requirements have been addressed.

We look forward to hearing from you in due course regarding our submission and to respond to any further questions and comments you may have.

Sincerely,

Juliet Abredu

(Corresponding author)
